# Synergistic platelet inhibition between Omega-3 and acetylsalicylic acid dose titration; an observational study

**DOI:** 10.1186/s12906-020-02990-9

**Published:** 2020-07-02

**Authors:** Harald Bagger, Mattias Hansson, Thomas Kander, Ulf Schött

**Affiliations:** 1 Institution of Clinical Science, Medical Faculty, Lund University, S-22185 Lund, Sweden; 2 Department of Anaesthesiology and Intensive Care, Skane University Hospital, S-22185 Lund, Sweden

**Keywords:** Coagulation, Acetylsalicylic acid, Omega-3

## Abstract

**Background:**

Omega-3 and acetylsalicylic acid (ASA) are two widely used “over-the-counter” drugs. Previous research has shown multiple electrode aggregometry (MEA) can detect ASA and varying Omega-3 platelet inhibiting effects. Synergistic platelet inhibiting effects of ASA and Omega-3 have been found using other methods than MEA. The aim of this study was to investigate the antiplatelet effects of Omega-3, and ASA synergism with MEA.

**Methods:**

Ten healthy male volunteers ingested Omega-3 (1260 mg/day) for 5 days. MEA was used to analyse platelet function before and after Omega-3 intake. Aggregation was initiated using three different agonists and measured as area under the curve (AUC): adenosine diphosphate (ADP), thrombin receptor activating peptide (TRAP) and arachidonic acid (ASPI). Two concentrations of ASA were dose titrated ex vivo to 2 out of 3 ASPI test cells in order to measure synergism between Omega-3 and ASA.

**Results:**

Following 5 days Omega-3 intake, ADP, TRAP and ASPI AUC did not change significantly. In vitro ASA before Omega-3 intake, reduced ASPI AUC < 30 U, indicating a strong platelet inhibiting effect. Below this AUC level, the 5 days Omega-3 intake increased ASPI-AUC with the ex vivo added low dose ASA (*P* = 0.02) and high dose ASA (*P* = 0.04).

**Conclusions:**

No synergism between ASA and Omega-3 was found using the MEA ASPI test. The surprising increase in ASPI-AUC following Omega-3 intake and ex vivo ASA suggest that there are methodological issuses with the MEA ASPI test.

**Trial registration:**

Trial registration ISRCTN78027929. Registered 19 May 2015.

## Background

Omega-3 fatty acids, primarily docosahexaenoic acid (DHA) and eicosapentaenoic acid (EPA), from fish oil are among the most frequently used dietary supplements, with 7.8% of U. S adults self-reporting frequent ingestion in 2012 [1]. Omega-3 has an established antiplatelet effect, and efforts have been made to examine through which specific mechanism(s) Omega-3 fatty acids affect platelet aggregation – with various results [2–5]. Dyerberg et al. [6] in 1979 suggested that high intake of Omega-3 fatty acids (fish oil) among Eskimos may cause increased bleeding. Definitive evidence is however nonexistent as to whether this antiplatelet effect on its own actually translates into increased clinical bleeding in patients [2, 7, 8]. A recent review identifies a platelet inhibiting effect of fish oil with no increased perioperative bleeding [9]. The platelet inhibiting effect of Omega-3 is probably weak, as the combination with clopidogrel does not to increase bleeding [10]. There are no reports on Omega-3 and stronger platelet inhibiting drugs. Omega-3 + warfarin does not increase bleeding [11]. There are no reports on Omega-3 and the new oral anticoagulants.

Aspirin (acetylsalicylic acid, ASA) is a widely used medication among adult patients for prevention of conditions such as cardiovascular disease, pain and inflammation. Between 2012 and 2015, more than 30% of U.S. adults aged 40 years and older self-reported taking low dose ASA for prevention of cardiovascular disease [12]. Considering the fact that the patient group most likely to frequently ingest supplementary Omega-3, namely patients at risk for cardiovascular disease, are also indicated for ASA treatment, a significant number of patients likely use both substances. ASA should not usually be withdrawn prior to surgery as it is considered to be a weak platelet inhibiting drug [13], with the exception of neurosurgery [14]. The effect can be measured with MEA, but a platelet inhibiting effect does not always transfer into an increased perioperative bleeding [15].

A synergistic or additive antiplatelet interaction between Omega-3 and ASA has been suggested by some studies [4, 16, 17]. A decrease in venous thromboembolism after total knee arthoplasty has been related to ASA+ Omega-3 synergism [18]. However, interactions between dietry supplements with antiplatelet effects and interactions with anticoagulation and antiplatelet drugs are not well studied [19].

The objective of the present study was to investigate the potential additional/synergistic effects of ASA + Omega-3 on platelet function with a point-of-care plateletet aggregometer, multiple electrode aggregometry (MEA), not studied before.

## Methods

This observational non-randomized, non-blinded screening study was approved by the Regional Ethical Review Board, Lund (registration number 2010/482) and was conducted in accordance with the World Medical Association Code of Ethics (Helsinki Declaration 1975). The manuscript was prepared in accordance with the STROBE guidelines for observational studies. Consent was given both orally and in writing.

Ten healthy, male volunteers (aged 21–29, median 23) were recruited. No females were studied as female gender affects platelet function/platelet count and MEA-results [20]. Also Omega-3 has a stronger platelet inhibitory effect on males [21]. Exclusion criteria were recent intake of alcohol, anticoagulant or antithrombotic medicine. Subjects were instructed to ingest a standard recommended dose (1260 mg) of two capsules of Omega-3 fish oil (Pharbio Omega-3 Forte®, Pharbio Medical International AB, Solna, Sweden) per day, one in the morning and one in the evening, for 4 days. Day 5, 2 capsules were ingested 2 h before sampling (see below). A total of ten capsules (12,600 mg) were ingested per volunteer during the study period. This daily dose represents an intake of 600 mg eicosapentaenoic acid (EPA), 400 mg docosahexaenoic acid (DHA), 60 mg docosapentaenoic acid (DPA) and 200 mg of other Omega-3. American Heart Association recommends at least 250 mg/day of EPA + DHA [22] and FDA not more than 2 g per day from dietary supplements (www.fda.gov).

### Blood sampling

Three ml of venous blood was drawn from an antecubital vein using a vacutainer system before and at the fifth day of Omega-3 treatment period. The volunteers rested 30 min before sampling and had had no stress or physical exertion during the morning, which can increase platelet aggregation [23]. Blood was collected in a 3.0 ml Hirudin blood tube (Roche Diagnostics GMbH, Mannheim; Germany). The hirudin sprayed within the blood tube exerts its inhibitory effect on thrombin, without interfering with physiological calcium levels [24]. The blood samples were stabilized at room temperature for 30 min, followed by MEA analysis within 3 h as recommended by the manufacturer [24] and Würtz et al. [25] to reduce test variability.

### Impedance aggregometry

MEA analysis was performed using Multiplate Analyzer® (Roche Diagnostics Scandinavia AB, Bromma, Sweden; V2.03.11). Through its 5 independent channels the Multiplate device is able to simultaneously measure the extent of platelet aggregation in each sample after the addition of different platelet agonists. Following the addition of platelet agonists, the change in electrical impedance between the two electrodes caused by the aggregation of platelets is detected. This change in electrical current is plotted in a graph over time and the results are expressed as area under the curve (AUC). The AUC represents the extent of platelet aggregation [26, 27].

Before analysis, 300 μl of prewarmed 9 mg/ml NaCl (B.Braun, Melsungen, Germany) was added to each test cell, followed by 300 μl of whole blood. The whole blood and NaCl were incubated and stirred for 3 min. Then 20 μl of platelet agonists were added to their designated test cells. Three different platelet agonists were used, I) Adenosine diphosphate (ADP), 6.5 μM, an agonist which binds to ADP receptors and activates platelets through the release of endogenous ADP from dense granules, II) thrombin receptor activating peptide (TRAP), (32 μM), a strong activator of platelets via the thrombin receptor, and III) ASPI (0.47–0.50 mM), which initiates platelet aggregation through the COX pathway [24]. COX is responsible for catalyzing the transformation of AA into thromboxane A2, a known potent activator of platelets [24].

Two concentrations of ASA were titrated to be added to two of three ASPI test cells, calculated to resemble real life blood concentrations with ASA treatment. 500 mg of ASA (Aspirin I.V. Bayer AG, Leverkusen, Germany) was diluted in 3 ml of sterile water to ca. 170 mg/ml. One concentration, termed high dose ASA, was diluted 10 times to 17 mg/ml. Another concentration, termed low dose ASA, was diluted 100 times to 1.7 mg/ml. Then 30 μl of low respectively high dose ASA was added to ASPI test cells with a final concentration, of 0,8 mg/ml and 0.08 mg/ml respectively. The concentration of 0.08 mg/ml closely resembles the estimated physiological concentration of 0.1 mg/ml following intravenous treatment of 500 mg ASA (the recommended treatment for acute myocardial infarction [28]. Intravenous (iv) aspirin ensures a better platelet inhibtion [29]. Our in vitro and ex vivo ASA protocol is more adherent to an iv approach.

Six minutes after addition of agonists the AUC of each test was recorded. ASPI-AUC was measured in three test cells: one without ASA and the other two in the test cells with added ASA. In total, the following 5 AUCs were analysed: ADP, TRAP, ASPI without ASA, ASPI with low and high dose ASA.

### Statistics

Sample size was calculated using G*Power version 3.1, (Heinrich-Heine-Universität Düsseldorf, Germany) and was based on previous data on MEA-analyses before and after Omega-3 intake [26, 27]. With an alfa error probability of 0.05 and a power of 0.9 the sample size had to be 8. We aimed at 10 volunteers to allow for sample failure.

The other statistical analyses were performed using GraphPad Prism version 8.3.0 (GraphPad Software Inc., La Jolla, CA). Gaussian distribution was tested using the D’Agostino & Pearson test and the Shapiro-Wilk test. As Gaussian distribution was found, statistically significant changes in AUC following Omega-3 intake were analysed using a paired T test. Significance level was set at *P* < 0.05. Potential outliers were analysed using the ROUT method.

## Results

**Fig. 1:**
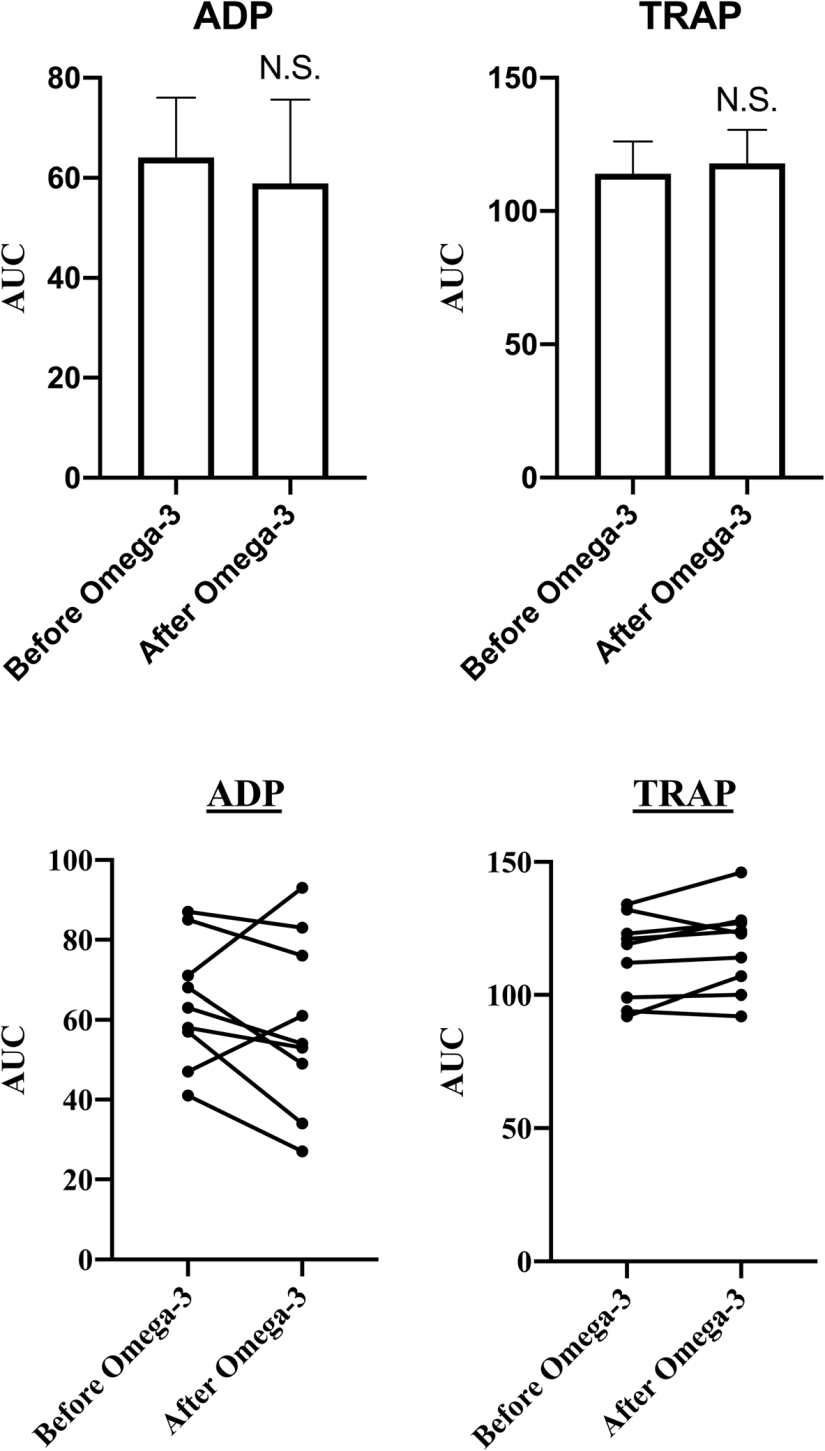
Results from multiple electrode aggregometry (MEA) before and after Omega-3 intake, using thrombocyte receptor activating peptide (TRAP) and adenosine diphosphate (ADP). Results are presented as mean with 95% confidence interval. N.S.: non-significant

**Fig. 2:**
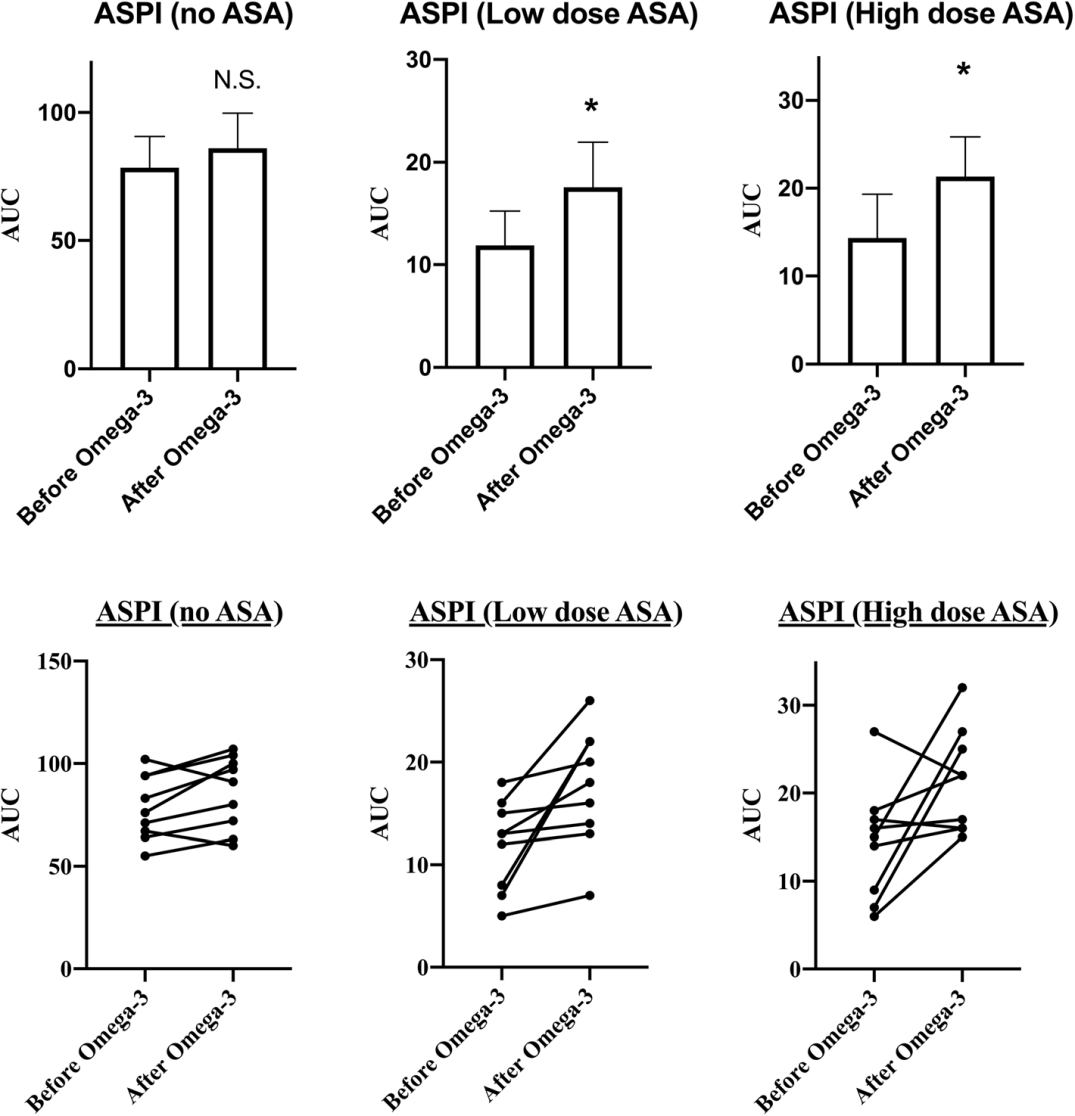
Results from multiple electrode aggregometry (MEA) before and after Omega-3 intake using arachidonic acid (ASPI) without acetylsalicylic acid (ASA), with low dose ASA and with high dose ASA. Note the different scales on the y-axes. *p = <0.05

One volunteer was excluded from the study due to poor compliance. In total, nine volunteers were included in the statistical analyses. There were no significant changes in the MEA ADP, TRAP or ASPI-assays after Omega-3 intake (Fig. 1 and Table 1). In vitro ASA before Omega-3 reduced ASPI AUC < 30 U, indicating a strong platelet inhibiting effect. Below this AUC level, both ex vivo added low dose ASA (*P* = 0.02) and high dose ASA (*P* = 0.04) increased ASPI-AUC on the 5th day of Omega-3 intake (Fig. 2 and Table 1). A corresponding borderline nonsignificant increase in ASPI AUC (*P* = 0.07) within normal reference range could also be detected from before Omega-3 and on the 5th day (without ASA) (Table 1), indicating some non-inhibitory effect of Omega-3 on the ASPI-test. No outliers were found.

**Table 1:** Results from multiple electrode aggregometry (MEA) assays

MEA-ASSAY	Reference AUC range	Mean AUC before Omega-3 (95% CI)	Mean AUC after Omega-3 (95% CI)	*P* – value
ADP	57–113	64 (52–76)	59 (42–76)	0.32
TRAP	84–128	114 (102–126)	118 (105–130)	0.15
ASPI with no ASA	71–115	78 (66–91)	86 (72–100)	0.07
ASPI with LOW DOSE ASA	N.A.	12 (9–15)	18 (13–22)	0.02
ASPI with HIGH DOSE ASA	N.A.	14 (9–19)	21 (17–26)	0.04

*AUC* area under the curve, *ADP* adenosine diphosphate, *TRAP* thrombocyte receptor activating peptide, *ASPII* arachidonic acid

## Discussion

The in vitro ASA effect on the MEA-ASPI prior to Omega-3 ingestion and the ex vivo ASA effect on the 5th day of Omega − 3 intake indicated a strong platelet inhibition with an ASPI AUC < 30 U. However, no additive or synergistic platelet inhibitory effect of 5 days peroral ingestion of Omega-3 was found using the MEA ASPI test.

The MEA ASPI test entails adding an AA agonist to the test cell blood sample, triggering platelet activation via the COX pathway. AA is a substrate of COX, which transforms AA into thromboxane A2, a potent platelet activator. It is a commonly held view that Omega-3 exerts its platelet function via inhibition of the AA-COX pathway. The basis for this view is that the Omega-3 fatty acids EPA and DHA competes with AA for COX, which might attenuate its action on AA. Also, following Omega-3 intake EPA and DHA are integrated into the phospholipid membrane, potentially at the expense of AA. This could also reduce AA-induced platelet aggregation [30]. If Omega-3 inhibits platelet aggregation via the AA-COX-axis, the ASPI AUC (representing AA induced platelet aggregation) would reasonably decrease following Omega-3 intake.

Our findings however contradict this view, as after Omega-3 intake the MEA ASPI AUC indicated an upward trend, although with no statistical significance, in the case of ASPI without ASA (*P* = 0.07). Significant increases in ASPI AUC after Omega-3 intake were seen with low dose ASA (*P* = 0.02) and with high dose ASA (*P* = 0.04) (Fig. 2). However, it must be stated that every individual value of ASPI AUC (without ASA) following Omega-3 intake was within normal MEA reference range [24, 26, 27], whereas some were below the reference value before Omega-3 intake (see below). Also, in the ASPI assays with both low and high dose ASA, all but 1 volunteer were below 30 U AUC (the exception having an AUC of 32 U in the ASPI+ASAx10 assay) following treatment. Less than 30 U ASPI AUC has been defined as the cut-off value for strong inhibition of COX-1 by ASA [31]. The fact that all but one AUC were still below this cut-off of strong inhibition, heavily indicate that Omega-3 did not actually reverse this inhibition, and that the significant increases in AUC of ASPI+low and high dose ASA may be related to test variability, intra-individual variation in test response at repeted testing [32]. Whole blood Multiplate testing with hirudin have a higher variability than citrate and asprin increase the test variability in hirudin anticoagulated ASPI-test [33]. With ongoing ASA treatment the MEA ASPI test has a coefficient of variation of 10% [34], but Pedersen et al. indentified a coefficient of variation (cv) of 8% increasing to 48% in the ASPI test before and after ASA treatment in healthy volunteers [35]. Such high ASPI test cvs have been corroborated by Peerschke et al. [33].

The lower ADP and ASPI AUC in some of the volunteers before Omega-3 intake may reflect unintentional over the counter drug intake or food/beverage intake with a similar effect, followed by a stricter regime during the study [32]. However only 2 of the volunteers increased in ADP response as compared to 7 in the ASPI response at the 5th day, hinting at a possible ASA/NSAID effect, even though the volunteers negated this. Measuring thromboxane could have identified ASA/NSAID effects [36].

Other studies examining the effects of Omega-3 intake using MEA have presented equivocal results. Kander et al. [26] found a significant decrease in ASPI AUC after adding Omega-3 in-vitro to blood from healthy volunteers. Mizia-Stec et al. [37] found a significant decrease of ASPI AUC in cardiovascular patients starting ASA and clopidogrel after percutaneous coronary inventions. A treatment group took Omega-3 – there were no differences in MEA (between Omega-3 patients and a control group without Omega-3 after 30 days in MEA (COL, TRAP, ASPI, ADP)), corroborating results of Watson et al. [10].

While our ASPI results do not necessarily support a procoagulant effect via the AA-COX pathway, they do indicate a lack of Omega-3 induced AA-COX antiplatelet effect. Recent studies also suggest this. Wada et al. [5] conducted a large experimental study comparing the effect of EPA versus AA on the COX pathway. EPA was found to be a very poor substrate for COX, suggesting that EPA has a limited ability to compete with AA as a COX substrate. Indeed, in a recent review article analysing multiple studies (including [5]) Wachira et al. [8] drew the conclusion that “there is little reason to believe that n-3 fatty acids affect platelet biochemistry primarily via the effects on the COX pathway”. Furthermore, while Gong et al. [4] found a synergistic effect on platelet aggregation between ASA and Omega-3 in mice, this was not affected by COX-1 knockdown. Aggregation was induced using ADP and collagen. Thus, while we in this study found no synergism or additive effect between ASA and Omega-3 in the AA-COX axis, such an effect may very well exist by other mechanisms. Also, Wachira et al. [8] suggests that metabolites of Omega-3 fatty acids produced through pathways other than the COX-axis could affect platelet function and thereby aggregation. Consequently, the highly dynamic and complex biochemistry of the multiple fatty acids present within platelets may affect our ability to isolate and determine the pro- or anti-platelet effect of individual fatty acid otherwise exerted in vivo [38]. Our results support the notion that Omega-3 works via other pathways than AA-inhibition.

In addition, several studies suggest that there could be other factors involved affecting the ASPI test and its AUC. In a recently published article, Ramström [39] investigated as to whether AA may cause lysis of blood cells, including platelets, and thereby affect ADP-dependent platelet activation. Indeed, increasing concentrations of AA was found to cause cell lysis and release of ADP, suggesting that platelet activation may be influenced by the presence of ADP in the ASPI test. However, in the present study no efforts were made to examine this phenomenon, suggesting that our results may have been subject to its potential effect. Thus, raised awareness of potential effect by AA on the ASPI test is needed.

Moreover, Christiansen et al. [40] recently published an article set to investigate as to whether risk variants of the AB0 locus among patients with stable coronary artery disease (CAD) may affect platelet activation and aggregation. Indeed, an increased platelet aggregation assessed by MEA was found to be associated with the risk variant rs495828. Although not examined in the present study, these findings suggest that possible risk variants of the AB0 locus among the included volunteers could have had an impact on our results. However, as the study population of Christiansen et al. predominantly consisted of male patients with stable CAD and a mean age of 65 years, their findings may not be relevant to ours.

Denis et al. [41] found that mature platelets contain components of the spliceosome, although earlier considered to be limited to nucleated cells. Thus, these findings may indicate that platelets are able to splice pre-mRNA in response to exogenous signaling. Furthermore, Evangelista et al. [42] support this hypothesis of preserved functions and found evidence of signal and time-dependent de novo synthesis of COX-1 in platelets, which may in part explain aspirin resistance. However, in the present study no sign of aspirin resistance among the included volunteers were observed. Also, a study conducted by Maree et al. [43] demonstrated that genetic variability in COX-1 may in part be responsible for the heterogeneous response observed in AA-induced platelets. In all, these studies indicate that platelets might contain preserved functions able to influence platelet aggregation, potentially explaining our surprising results.

The present study adds to the complexity of determining the effect of Omega-3 fatty acids on platelet aggregation. Of note, 5 out of 9 volunteers were below MEA ADP AUC reference range following Omega-3 intake, in line with the decreasing trend. In all, these findings are consistent with previous studies; in a metanalysis of 15 studies, Gao et al. [44] found a significant reduction in ADP-induced aggregation. As previously mentioned, the activation of platelets is complex and several components and pathways are thought to be involved. ADP is an important agonist which induces platelet aggregation through the membrane-bound P2Y_12_-receptor by indirectly increasing the cytosolic calcium-level (Ca^2+^) and thereby enable the complex formation of GpIIb/IIIa [45]. However, some studies show the contrary, indicating a negligible effect on ADP-induced platelet aggregation in the presence of Omega-3 fatty acids [27, 46].

Perhaps more advanced laboratory technology should be used to study Omega-3 effects on platelets. Omega-3 can reduce the von Willebrand factor (shear dependant platelet activator) [47]. We have previously studied Omega-3 effects with an automatic flow chamber technique (Cellix™) with high shear stress, but failed to detect any platelet inhibiting effect [48]. Cohen et al. used electrophoretic quasi-elastic light scattering technology (EQELS) technology to study Omega-3 effects and found an increased negative resting platelet charge, e.g. decreased response to AA platelet activation [49]. They also used template bleeding time that increased with increasing doses from 1 to 8 g Omega-3. Interesting there were no changes in light transmission aggregometry, not even in patients on ASA/clopidogrel, corroborating our results that platelet aggregometry is not the adequate technique. The Omega-3 fraction EPA but not DHA can reduce platelet volume, an early sign of reduced platelet aggregation [50].

This study has several limitations. Only healthy young men were recruited as volunteers. As hypertriglyceridemia and coronary arterial disease have been shown to positively affect platelet responsiveness to Omega-3 supplementation [44], volunteers should have been more physiologically representative of the relevant patient population. While every volunteer denied recent intake of ASA or NSAIDs, intake prior to enrolment in the study likely varied. Measurement of thromboxane levels in blood or urine could have identified this, but needs special laboratory resources [36]. Also, convincing evidence from several studies suggest that diet may have considerable influence on platelet function, and thereby risk of cardiovascular disease as well [51–54]. In the current study the volunteers were not instructed to report dietary habits. However, in a recent study conducted by Krekels et al. [55] no significant effect on platelet aggregation after smoking, coffee, high-fat meals or physical exercise was observed. Thus, future studies examining as to whether diet may affect platelet aggregation, when measured by methods such as MEA are needed. Furthermore, the great inter-individual variation of volunteers in response to Omega-3 treatment, as is evident in the individual response graphs in Figs. 1 and 2, makes clear interpretation of results difficult. For instance, consider the ADP individual response graph with two volunteers strongly deviating from the otherwise clear trend of reduction, thus possibly skewing results. A greater sample size would limit this susceptibility and would increase statistical power.

Another limitation of this study is the fact that the 5-day Omega-3 treatment period might have been insufficient for Omega-3 to actually exert its platelet effects, although signifcant increases in plasma and red cells Omega-3 fatty acid concentrations can be meassured already at day 7, but increasing over time [56]. Intravenous administration of Omega-3 is rapidly taken up by platelets (4 h) and by erythrocytes (ERC) (improves deformability and blood flow [47]) after 48 h [57]. In vitro incubation with Omega-3 reduces thromboxane B2, prostaglandins F2,E 2 and D2 production from platelets already after 5 min [58]. Cao et al. [59] found an ingestion period of 8–20 weeks necessary to achieve an Omega-3 index (measuring total Omega-3 fatty acid concentration in plasma phospholipids and ERC membranes) recommended by von Schacky et al. for acardioprotective effect [60]. Different Omega-3 purified fractions also inhibit platelet aggregation with different agonists after varying oral intake periods. Platelet aggregation with DHA (6 g/day) was reduced already after 6 days (collagen/ADP), but with EPA only platelet aggregation to collagen could be detected so early; for ADP it took 4 weeks of EPA intake to detect a reducing effect [60].

Bagge et al. [26] found a significant change in MEA ADP AUC following 7 days of daily 1260 mg Omega-3 ingestion in a prospective pilot study. However, in a follow-up study with 10 days of daily 2520 mg ingestion, no significant results were found [48]. While Cao et al. did not use MEA (unlike Bagge et al.), the far more direct method of measuring Omega-3 levels used by Cao et al. make their study more relevant in terms of discussing adequate Omega-3 treatment period for future studies. All in all, it is possible that significant results found after a treatment period shorter than that recommended by Cao et al. are merely chance findings that should be treated with caution. Recent research use higher doses of Omega-3 for longer periods and very advanced laboratory methods to evaluate cellular and antiinflammatory effects on cardiovascular health [60]. Although Omega-3 effects on platelets can be seen better in healthy volunteers, patients with risk factors should be studied – with higher doses [61, 62].

## Conclusions

No synergism between ASA and Omega-3 was found using the MEA ASPI test. The surprising increase in ASPI-AUC following Omega-3 intake and ex vivo ASA suggest that there are methodological issuses with the MEA ASPI test. Other platelet laboratory methods should be used to study Omega-3 effects on platelet function.

## Data Availability

Data will be available on reasonable request.

## Abbreviations

ASA: Acetylsalicylic acid
MEA: Multiple electrode aggregometry
AUC: Area under the curve
ADP: Adenosine diphosphate (ADP), t
TRAP: Thrombin receptor activating peptide
ASPI: Arachidonic acid
DHA: Docosahexaenoic
EPA: Eicosapentaenoic acid
COX: Cyclooxygenase
AA: Arachidonic acid
